# Unusual Five Copies and Dual Forms of *nrdB* in “*Candidatus* Liberibacter asiaticus”: Biological Implications and PCR Detection Application

**DOI:** 10.1038/srep39020

**Published:** 2016-12-13

**Authors:** Zheng Zheng, Meirong Xu, Minli Bao, Fengnian Wu, Jianchi Chen, Xiaoling Deng

**Affiliations:** 1 Guangdong Province Key Laboratory of Microbial Signals and Disease Control, Citrus Huanglongbing Research Laboratory, South China Agricultural University, Guangzhou, Peoples’ Republic of China; 2 San Joaquin Valley Agricultural Sciences Center, United States Department of Agriculture–Agricultural Research Service, Parlier, California, United States of America

## Abstract

“*Candidatus* Liberibacter asiaticus” (CLas), a non-culturable α-proteobacterium, is associated with citrus Huanglongbing (HLB, yellow shoot disease) currently threatening citrus production worldwide. Here, the whole genome sequence of CLas strain A4 from Guangdong of China was analyzed. Five copies of *nrdB*, encoding β-subunit of ribonucleotide reductase (RNR), a critical enzyme involving bacterial proliferation, were found. Three *nrdB* copies were in long form (*nrdB*^*L*^, 1,059 bp) and two were in short form (*nrdB*^*S*^, 378 bp). *nrdB*^*S*^ shared >99% identity to 3′ end of *nrdB*^*L*^ and had no active site. Sequences of CLas *nrdB* genes formed a distinct monophyletic lineage among eubacteria. To make use of the high copy number feature, a *nrdB*-based primer set RNRf/RNRr was designed and evaluated using real-time PCR with 262 HLB samples collected from China and USA. Compared to the current standard primer set HLBas/HLBr derived from the 16S rRNA gene, RNRf/RNRr had Ct value reductions of 1.68 (SYBR Green PCR) and 1.77 (TaqMan PCR), thus increasing the detection sensitivity three-fold. Meanwhile, RNRf/RNRr was more than twice the stability of primer set LJ900f/LJ900r derived from multi-copy prophage. The *nrdB*-based PCR thereby provides a sensitive and reliable CLas detection with broad application, especially for the early diagnosis of HLB.

“*Candidatus* Liberibacter asiaticus” (CLas), a phloem-limited α-proteobacterium, is associated with citrus Huanglongbing (HLB, yellow shoot disease, also known as citrus greening disease) that is devastating citrus production worldwide^1^,^2^. No effective cure for HLB is currently available. Management of HLB depends on excluding CLas from citrus-producing regions though use of regional quarantines, pathogen-free nursery stocks, removal of infected trees, and control of vectors, e.g. the Asian citrus psyllid (ACP, *Diaphorina citri*). Knowledge about CLas biology plays critical roles for development of novel, effective HLB control strategies. Yet, study of this bacterium has been difficult due to the inability to culture it *in vitro*.

Recent developments in bacterial whole genome sequencing through next generation sequence (NGS) technology have opened a new venue for research in non-culturable plant pathogenic bacteria. We recently sequenced the whole genome of CLas strain A4 from Guangdong, China where HLB was first described^3^,^4^. Analyses of genome sequence of the A4 strain has led to discovery of a CRISPR/cas system and dominant single prophage phenomenon in CLas strains in China^5^. We also observed several large (>300 bp) DNA duplications in the strain A4 chromosome. One of them was identified as ribonucleotide reductase (RNR) β-subunit gene, *nrdB*. RNR is a key enzyme for converting ribonucleotides to deoxyribonucleotides, the precursors of DNA synthesis and repair, which is under strict regulation during cell proliferation^6^,^7^,^8^. RNR is also an important target for development of antibacterial drugs^8^. There have been extensive studies on RNR and its genes in model bacteria^6^,^7^,^8^. A database dedicated for RNR research has been established^9^. Currently, no information about CLas RNR has been published, except for a brief mention of a partial RNR gene sequence in PCR detection^10^.

Detection of CLas mainly relies on PCR technologies involving the use of specifically designed primer sets based genomic DNA sequences, mostly the 16S rRNA gene. Examples are primer set OI1/OI2c for standard PCR^11^ and primer set HLBas/HLBp/HLBr for TaqMan real-time PCR^12^. The chromosome of CLas has three copies of the 16S rRNA gene^13^. One strategy for further improvement of PCR detection is to identify and target genes with >3 copies. The proof of concept has recently been achieved in PCR detection of *Spiroplasma citri*, causing citrus stubborn disease by targeting multi-copy phage genes^14^. In CLas, a phage-based primer set (LJ900f/LJ900r) has been developed and tested^15^. However, recent investigation showed that CLas prophages and their sequences were highly variable including the absence of prophage^5^,^16^, which could impede detection reliability or accuracy. The high copy number *nrdB* provides an ideal target for sensitive detection of CLas.

The aims of this research were: (1) characterize *nrdB* in CLas based on available RNR information and bacterial genome sequences and predict its possible biological role; (2) elucidate phylogenetic relationships of CLas among eubacteria based on *nrdB* DNA and amino acid sequences; and (3) evaluate the use of a *nrdB*-based primer set for improvement of CLas detection, with comparisons made to existing PCR primers such as the 16S rRNA gene-based primer set HLBas/HLBr and the prophage sequence-based primer set LJ900f/LJ900r.

## Results

### Identification of multiple-copy regions in A4 genome

**Figure 1:**
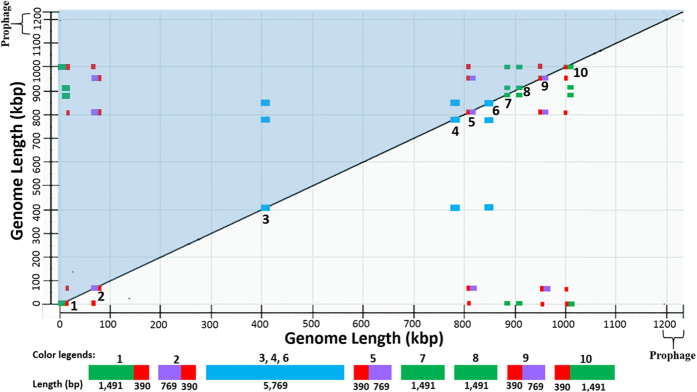
Visualization of repeat regions in the genome sequence of “*Candidatus* Liberibacter asiaticus” (CLas) strain A4 (CP010804). The dot-matrix map was created by self-comparison through BLAST program available in National Center for Biotechnological Information. Genome length was marked in both X- and Y-axis with the prophage region identified. The upper-left diagonal (in blue shadow) shares the same information as the bottom-right diagonal. Examination on one diagonal (e.g. the bottom-right) reveals ten repeat regions on the diagonal line labeled with numbers accordingly. Sequences sharing >99.9% similarities (repeats) among the ten regions are marked with the same color. The red color sequence (390 bp) has the higher copy number of five (Region 1, 2, 5, 9, and10). Region 3, 4, and 6 are *rrn* operon in blue.

As shown in Fig. 1, ten repeat regions were detected in the A4 genome by Dot Matrix analysis. Examination of the retrieved sequences revealed that regions 3, 4 and 6 were identical DNA sequences of 5,769 bp, each containing the genes of 16S, 23S and 5S rRNAs or the *rrn* operon (Supplementary Table S1). The other seven regions were sequences of three different sizes: 1,881 bp for region 1 and 10, 1,059 bp for regions 2, 5, and 9, and 1,491 bp for regions 7 and 8. Results of sequence alignments showed that regions 1, 2, 5, 9, and 10 contained a common 390-bp sequence (red in Fig. 1); regions 1, 7, 8, and 10 contained a common 1,492 bp sequence (green in Fig. 1); and regions 2, 5, and 9 contained a common 769 bp sequence (purple in Fig. 1). Genes or open reading frames (ORFs) corresponding to each region were listed in Supplementary Table S1.

### Characterization of CLas *nrdB*

**Figure 2:**
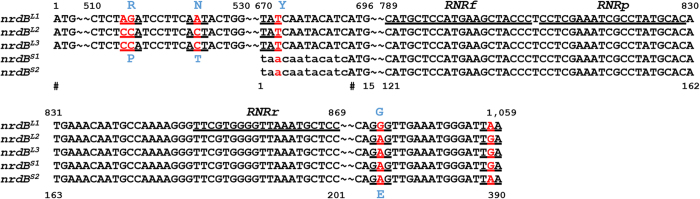
Alignment of five ribonucleotide reductase β-subunit gene (*nrdB*) related sequences in “*Candidatus* Liberibacter asiaticus” strains A4 (CP010804) showing single nucleotide polymorphisms (SNPs) and TaqMan PCR primer and probe designs. Position numbers are listed above or under are the sequence; SNPs are identified in red with corresponding codon underlined and amino acids indicated above or under in blue. Sequence of TaqMan primers (RNRf/RNRr) and probe (RNRp) are underlined; “~~”represents omitted identical nucleotides; # indicates initial position of *nrdB*^*S*^or *nrdB*^*L*^.

**Figure 3:**
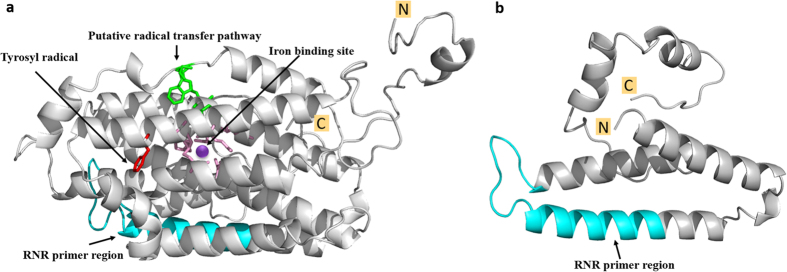
Predicted 3-D structures of *nrdB*^*L1*^ (long form) and *nrdB*^*S1*^ (short form) of ribonucleotide reductase β-subunit. **(a**) *nrdB*^*L*^ and (**b**) *nrdB*^*S*^. The iron binding residues in pink centered by a purple dot (binding site), the tyrosyl radical in red, the putative radical transfer pathway in green. The regions targeted by primer set RNRf/RNRr were highlight in cyan. All conserved residues and model was generated using Phyre server^29^. The final refinement of all 3-D structure figures were made using the Pymol Molecular Graphics System (v1.7.6).

Since the 390 bp sequence was repeated five times (the highest) in the CLas genome, the 390 bp-containing sequences, i.e. region 1, 2, 5, 9, 10 (Fig. 1) were selected for further study. In region 1 and 10, 378 of the 390 bp formed ORFs CD16_00035 and CD16_04445, respectively (Supplementary Table S1). In region 2, 5, and 9, the whole 1,059 bp formed ORFs CD16_00300, CD16_03625, and CD16_04230, respectively (Supplementary Table S1). All five sequences were annotated as *nrdB* encoding the β-subunit of RNR Class Ia (EC 1.17.4.1), two (CD16_00035 and CD16_04445) in short form (*nrdB*^*S*^, 125 amino acids) and three (CD16_00300, CD16_03625, and CD16_04230) in long forms (*nrdB*^*L*^, 352 amino acids) (Table 1). Note that 12 bp at the 5′ end of the 390-bp sequence were not part of *nrdB*^*S*^ (Fig. 2). *nrdB*^*S1*^and *nrdB*^*S2*^ had a SNP at position 389, part of the synonymous stop codons. Five SNPs were found among *nrdB*^*L1*^, *nrdB*^*L2*^, and *nrdB*^*L3*^ without causing frame shifts (Fig. 2). Conserved domain analysis indicated the long nrdB^L^ protein (352-aa) contained a diiron center (ion binding site), the tyrosyl radical, a putative radical transfer pathway and a dimer interface (polypeptide binding site) (Fig. 3). No iron binding site was identified on the short nrdB^S^ protein (125-aa) as shown in the predicted 3-D structures (Fig. 3).

**Figure 4:**
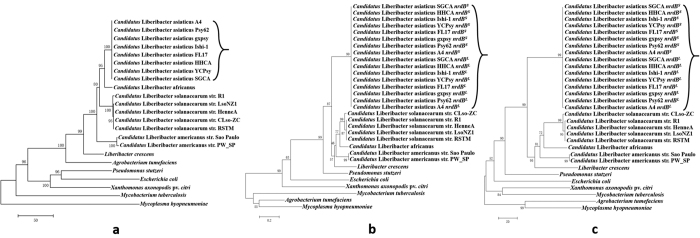
Phylogenetic trees of “*Candidatus* Liberibacter asiaticus” related to other bacteria. (**a**) Based on the DNA sequence of 16S rRNA genes. (**b**) Based on the amino acid sequence of ribonucleotide reductase gene β-subunit *nrdB*. (**c**) Based on the DNA sequence of *nrdB*. The CLas cluster was labeled by the right brace.

BLASTn search against all published CLas genome sequences revealed that all CLas strains had the same number of nearly identical *nrdB* genes (both *nrdB*^*S*^ and *nrdB*^*L*^) (Table 2), except for the CLas strain SGCA5, which could be due to the influence of *de novo* assembly^17^ that dropped out repeat sequences because reassembly using A4 sequence as a reference showed the same five *nrdB* genes (unpublished data). The copy number of *nrdB* in CLas was much higher (five) than all the non-CLas Liberibacters, as well as those of other bacterial species (Table 2). Phylogenetic trees of selected representative bacteria based on 16S rRNA gene, amino acid sequence and DNA sequence of *nrdB* gene are shown in Fig. 4. In all three trees, Liberibacters were clustered together. Within Liberibacters, CLas clustered together, demonstrating the monophyletic lineage of CLas based on *nrdB* gene as that of the 16S rRNA gene. It is, however, noted that based on 16S rRNA gene tree, *Agrobacterium* was closely related to Liberibacters. This was not the case in the *nrdB* gene tree.

### Specificity of RNR primer set

Primer set RNRf/RNRr was designed based on the 390 bp repeats in the CLas genome (Fig. 2; Table 3). BLASTn search (word size = 16) using RNRf/RNRr primer sequences as queries against the GenBank nr/nt database that contained >1,000 bacterial genome sequences returned hits strictly to the RNR gene of CLas. PCR of DNA samples extracted from two healthy citrus plants and one CLas-free psyllid reared in our laboratory showed no amplification with primer set RNRf/RNRr by SYBR Green real-time PCR. The melting point of RNRf/RNRr amplicon was at 81.50 °C.

### Evaluations among RNRf/RNRr, HLBas/HLBr, and LJ900f/LJ900r

**Figure 5:**
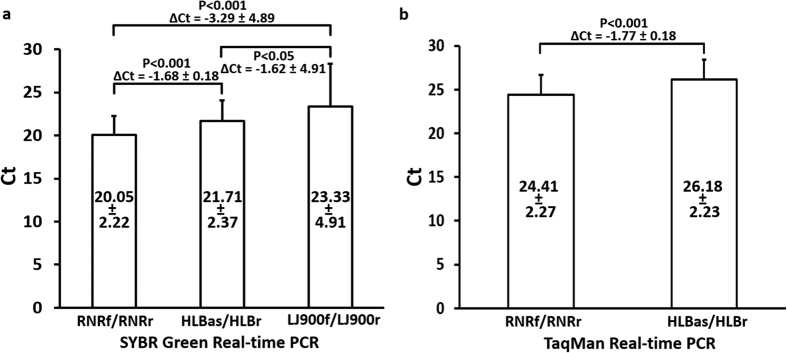
Comparisons of PCR detection sensitivities on “*Candidatus* Liberibacter asiaticus” using 57 samples from China (34) and USA (23) among primer sets RNRf/RNRr (*nrdB*-based), HLBas/HLBr (16S rRNA gene-based), and LJ900f/LJ900r (prophage-based). (**a**) SYBR Green real-time PCR. (**b**) TaqMan Real-time PCR. Numbers within each bar box are mean Ct values with standard deviation. P values were calculated based on independent-sample T-test. All qPCR assays were performed on the ABI real-time PCR system with the same regent kit (Universal PCR Master Mix, Applied biosystems).

A total of 57 CLas samples collected from China and USA were selected for primer set evaluations (Fig. 5). Sensitivity comparisons were performed simultaneously by SYBR Green real-time PCR format (all three primer sets) and TaqMan real-time PCR (RNRf/RNRr and HLBas/HLBr). As shown in Fig. 5, mean Ct values were 20.05 for RNRf/RNRr, 21.71 for HLBas/HLBr, and 23.33 for LJ900f/LJ900r. Standard deviations from RNRf/RNRr (2.22) and HLBas/HLBr (2.37) were smaller than that from LJ900f/LJ900r (4.91), suggesting higher sequence variations of CLas prophages than those of the conserved 16S rRNA gene and *nrdB*. Mean Ct differences between RNRf/RNRr and HLBas/HLBr were significant P < 0.001 in both SYBR Green PCR and TaqMan PCR formats, with ΔCt being −1.68 ± 0.18 for SYBR green PCR and −1.77 ± 0.18 for TaqMan PCR. These represent >3 fold increase of sensitivity based on the ΔCt method^18^. Differences between RNRf/RNRr and LJ900f/LJ900r and between HLBas/HLBr and LJ900f/LJ900r were also significant at P < 0.05 level.

### Evaluation on RNRf/RNRr with field samples from China and USA

A total of 262 DNA samples extracted from CLas infected plants and psyllids in seven provinces in China and three states in USA were tested with SYBR Green real-time PCR format (Table 4). Overall, there was a significant difference between the Ct values of RNRf/RNRr and HLBas/HLBr (P < 0.0001), although variations existed from location to location in both countries. The largest P value in China was from Guangxi Province and the largest P value in USA was from Florida. However, in all cases, P values were <0.05 and ΔCt were negative within a range from −1.36 to −1.75 (Table 4). In addition, the RNRf/RNRr qPCR assays on three different qPCR systems (ABI system, MJ system, and CFX system) also showed the robust of RNRf/RNRf on detection of CLas (Table S2).

## Discussion

The inability to culture CLas *in vitro* limits the use of traditional *in vitro* culture-based methodologies to study its biology. Genome sequence analyses in this study provided the first insight into an RNR gene of CLas and reveal previously unknown properties of the bacterium. According to model studies, RNRs are divided into three classes (Classes I, II, and III), largely based on their interaction with oxygen and the way in which they generate their tyrosyl racdical^19^. The CLas *nrdB* described in this study belongs to Class Ia, that is exclusively oxygen-dependent^8^, implying an aerobic lifestyle of CLas. This is the first report on oxygen usage status of CLas, which will benefit future efforts on *in vitro* cultivation of the bacterium.

Typically, bacterial RNR genes are arranged in an operon. Class Ia RNR genes form *nrdAB*, where *nrdA* encodes RNR α-subunit, and *nrdB* encodes RNR β-subunit. This does not seem to be the case in CLas, where both *nrdA* and *nrdB* are dispersed separately in the bacterial genome (Table 1). Examinations of neighboring regions of each *nrdB* gene revealed no RNR gene homologs with the exception of *nrdB*^*L2*^ (Supplementary Table S3). Upstream of *nrdB*^*L2*^ is *RibF* (Supplementary Table S3), encoding a riboflavin biosynthesis protein similar to that of *nrdI* in the Class Ib operon *nrdHIEF* where *nrdH* encodes a glutaredoxin-like protein, *nrdI* encodes a flavorotein, *nrdE* encodes RNR α-subunit, and *nrdF* encodes RNR β-subunit^20^. It was also reported that in *Mycobacterium tuberculosis,* RNR subunit genes were not arranged in an operon^21^. Interestingly, both CLas and *M. tuberculosis* are nutritionally fastidious intracellular pathogens. The HLB associated CLas is not cultivable. The slow growing *M. tuberculosis* causes human tuberculosis.

The most intriguing finding from this study is that CLas has five copies *nrdB*, three in a long form designated *nrdB*^*L*^ and two in a short form designated *nrdB*^*S*^, along with a single *nrdA* (Table 1). As shown in Table 2, among the known Liberibacter genomes, only CLas has multiple copies of RNR genes. Although it is common to find multiple RNR classes within a single bacterial species^8^, only a few cases of *nrd* gene direct duplication have been reported. For example, *M. tuberculosis* has a second class Ib-like subunit gene^21^ and *Sreptococcus pyogenes* has two clusters of class Ib genes, *nrdHEF* and nrdF*I*E*^22^. In both cases, the duplicated genes show significant variations at the level of DNA sequences (<71% identity). In this study, the sequences of three *nrdB*^*L*^ are almost identical and the two *nrdB*^*S*^ are nearly identical. The common regions between *nrdB*^*L*^ and *nrdB*^*S*^ are also identical. These indicate that the *nrdB* gene duplication events are recent.

Duplication of RNR genes has been shown to be important for bacterial proliferation. As in the cases of *M. tuberculosis* and *S. pyogenes*, the two different *nrd* genes allowed bacterial growth under different growth environments^21^,^22^. Along this direction, the *nrdB* duplication in CLas could be related to its environmental adaptation and likely by increasing functional dosage^23^. Although more evidence is needed, it will be of interest to study if this possible dosage effect could be linked to the current dominance of CLas in HLB. In Brazil, both CLas and CLam were reported to be associated with HLB^24^. However, as observation continued, the population of CLas increased whereas the population of CLam decreased^25^,^26^.

It is noted that *nrdB*^*S*^ has no active site (Fig. 4). Its biological role(s) could be an interesting topic. In early research, a strain of *Escherichi coli* (C600) was found to have two forms of β-subunit of RNR, one was a full length and functional β-polypeptide, the other was a truncated and non-functional β’-polypeptide^27^. In a model RNR structure of α2β2, there could be two possible homodimeric β-subunits (ββ and β’β’) and one heterodimeric β-subunit (ββ’). The heterodimeric β-subunit was found to conform to a half-site reactivity, which might be involved in regulation of enzyme activity. In this regard, we speculate that the non-functional short form *nrdB*^*S*^ could be used at the transcriptional level to generate a heterodimer as part of the RNR regulation in CLas proliferation.

While *in silico* genome sequence analyses of RNR genes only provide information for understanding CLas biology, the high copy number and conserved feature of *nrdB* was explored for CLas detection. The use of primer set HLBas/HLBr along with a hybridization probe (TaqMan PCR) has been regarded as a standard protocol for CLas detection. However, problems arise when high Ct values, e.g. Ct = 30 or higher, are encountered. This situation is commonly encountered when testing citrus trees for the presence of CLas, especially for symptomless or atypical symptom samples. The available RNRf/RNRr PCR detection system provides a remedy. First, as HLBas/HLBas, RNRf/RNRr was also based on the highly conserved gene. This assured the reliability of CLas detection, in contrast to the prophage-based primer set Lj900f/LJ900r (Fig. 5). In fact, the universal presence of RNR gene has been recommended as a key target for phylogeny research of viruses that lack ribosomal RNA genes^28^; and second, the RNRf/RNRr locus has five copies, higher than the three copies of the 16S rRNA gene. This means more initial targets are available for PCR leading to increased sensitivity of detection. As demonstrated in Fig. 5, RNRf/RNRr PCR is at least three times more sensitive than HLBas/HLBr PCR in both SYBR green and TaqMan formats. In this study, the robust of RNRf/RNRrqPCR assays were also confirmed on three different real-time PCR system, although greater sensitivity of RNR primers was showed on both ABI system and MJ system rather than on CFX system (Table S2).

In summary, through genome sequence analyses, we discovered that CLas had five copies of RNR β-subunit gene *nrdB*. CLas *nrdB* has both long and short forms that could play a role in the RNR regulation in the bacterial proliferation. Phylogenetically, all CLas *nrdB* genes clustered together, forming a stable evolutionary lineage, as that of the 16S rRNA gene. The high copy number and conserved feature of *nrdB* provide a foundation for being used in sensitive and reliable detection of CLas. Primer set RNRf/RNRr has been developed and tested. The detection system is recommended for use to resolve CLas detection issue when the primer set HLBas/HLBr encounters border line Ct for interpretation.

## Materials and Methods

### Bacterial genome sequences and strains

The whole genome sequence of CLas strain A4 that originated from an HLB citrus tree in Guangdong of China (CP010804)^3^ was used for DNA/gene copy evaluation. All bacterial genome sequences were downloaded from GenBank database (v211.0) hosted by the National Center for Biotechnology information (NCBI) (Table 2). Field strains were collected for population study. A CLas strain was represented by DNA extracted from an infected leaf sample of citrus (*Citrus* sp.) or periwinkle (*Catharanthus roseus*) or an individual ACP. Samples were from seven provinces (Guangdong, Guangxi, Yunnan, Fujian, Jiangxi, Zhejiang and Hainan) in China and three states (Florida, Texas and California) in USA (Table 4). DNA were extracted by with E. Z. N. A. HP Plant DNA Kit (OMEGA Bio-Tek Co., Guangdong, China) or DNeasy Plant Kits (Qiagen Inc., Valencia, CA) for plant samples, and TIANamp Genomic DNA Kit (Tiangen Biotech Co., Beijing, China) or DNeasy Blood & Tissue Kit (Qiagen Inc., Valencia, CA) for individual psyllid.

### Identification of *nrdB* and *in silico* characterization

The CLas strain A4 genome sequence was self-compared using the BLASTn program with the word size set at 128-bp with the web service of NCBI. The result was visualized with the Dot-Matrix option. DNA sequence regions with highest number of repeats were retrieved. The genetic nature of DNA sequences was characterized according to genome annotation, assisted by BLAST search against the NCBI conserved domain database (CDD, v3.14). Since the identified DNA sequences were longer than the annotated genes, only gene sequences were downloaded and used for analyses. Protein structure analyses were initially carried out with Phyre server (http://www.sbg.bio.ic.ac.uk/~phyre2/html/page.cgi?id=index) using a profile-profile alignment algorithm^29^. Final 3-D structures were made using Pymol Molecular Graphics System (v1.7.6, Schrödinger LLC).

For phylogenetic studies, all published CLas and selected bacterial species representing major bacterial groups were used (Table 2). DNA and amino acid sequence of *nrdB* were retrieved according to genome annotation or from the ribonucleotide reductase database (v0.901)^9^. The total number of *nrdB* gene in each genome was directly counted from the genome annotation and further confirmed by similarity searching the bacterial genome with the corresponding *nrdB* sequence. DNA sequences of 16S rRNA genes were downloaded from NCBI GenBank nucleotide database (Genbank version 211.0). Phylogenetic trees were constructed using the Neighbor-joining method with MEGA 6.0^30^.

### Primer/probe designs and PCR experiments

CLas nrdB sequences were aligned through the Clustal Omega software^31^. Common regions across all nrdB sequences were identified and used to design PCR primers and TaqMan probe sequences with Primer 3 software^32^ (Table 3). Primer and probe sequence specificity were checked through BLASTn against the GenBank nucleotide database (Genbank version 211.0). The TaqMan probe was synthesized by labeling the 5′-terminal nucleotide with 6-carboxy-fluorescein (FAM) reporter dye and the 3′-terminal nucleotide with Black Hole Quencher (BHQ)-1 (Table 3) through a commercial source. Primers of HLBas/HLBr and HLBp and LJ900f/LJ900r were synthesized according to the original publication^12^,^15^.

Both SYBR Green and TaqMan real-time PCR formats were used in this study. The SYBR Green real-time PCR assays were performed in three different real-time PCR systems. In the USA, MJ Research DNA Engine opticon 2 system (MJ; MJ Research Inc), and Applied Biosystems StepOnePlus™ Real-Time PCR Systems (ABI; Applied Biosystems, Foster City, CA, US) were used. In China, the CFX Connect Real-Time System (Bio-Rad, Hercules, CA, USA) was used. The TaqMan real-time PCR assays were only performed in the Applied Biosystems StepOnePlus™ Real-Time PCR Systems.

Real-time PCR procedures were essentially referenced to that of Li *et al*.^12^. For SYBR Green real-time PCR, the reaction mixture contained 10 μl of iQ™ SYBR^®^ Green Supermix (Bio-Rad) or Fast SYBR^®^ Green Master Mix (Applied Biosystems) or Bestar^®^ qPCR Master Mix (DBI^®^ Bioscience), 1 μl of DNA template (~25 ng), 0.5 μl of each forward and reverse primer (10 μM) in a final volume of 20 μl with the following procedure: 95 °C for 3 min (MJ and CFX) or 95 °C for 20 s (ABI), followed by 40 cycles at 95 °C for 10 s (MJ) or 95 °C for 3 s (ABI) or 95 °C for 10 s (CFX, Bio-Rad) and 60 °C for 30 s (MJ and CFX) or 60 °C for 3 s (ABI). The fluorescence signal was captured at the end of each 60 °C step, followed by a melting point analysis.

For TaqMan^®^ real-time PCR, the reaction mixture contained 10 μl of TaqMan^®^ Fast Universal PCR Master Mix (2X) (Applied Biosystems), 1 μl of DNA template (~25 ng), 0.2 μl of TaqMan^®^ probe (5 μM), 0.4 μl of each forward and reverse primer (10 μM) in a final volume of 20 μl with the following procedure: 50 °C for 2 min, then 95 °C for 20 s, followed by 40 cycles at 95 °C for 3 s and 60 °C for 30 s. The fluorescence signal was captured at the end of each 60 °C step. The data were analyzed using Opticon Monitor™ software (MJ Research), StepOnePlus™ Software v2.3 (Applied Biosystems) and Bio-Rad CFX Manager 2.1 software with automated baseline settings and a manually set threshold at 0.1. Amplicons were quantified using standard curves established based on ten-fold serial dilutions of the CLas-infected citrus plant total DNA in triplicate.

For evaluation of differences among primer sets of RNRf/RNRr, HLBas/HLBr, and LJ900f/LJ900r, 34 CLas samples from China and 10 CLas samples from USA were used (Table 3). The SYBR green real-time PCR format was used to for primer set evaluations. Since HLBas/HLBr-HLBp (TaqMan real-time PCR format) was popularly used, RNRf/RNRr-RNRp was also used. To substantiate the evaluation results, a total of 262 CLas samples collected from China and USA (Table 4) were tested with SYBR green format.

### Statistical analysis

PCR results (Ct values) among different primer sets were evaluated by independent-sample T test. All tests were performed using the SPSS Statistic package (v19.0, IBM, Armonk, New York, U.S.). Sensitivity increase (R) between RNRf/RNRr and HLBas/HLBr was calculated through the ΔCt method^18^, i.e. R = 2^−ΔCt^, ΔCt = Ct (RNRf/RNRr)–Ct (HLBas/HLBr).

## Additional Information

**How to cite this article:** Zheng, Z. *et al*. Unusual Five Copies and Dual Forms of *nrdB* in “*Candidatus* Liberibacter asiaticus”: Biological Implications and PCR Detection Application. *Sci. Rep.* **6**, 39020; doi: 10.1038/srep39020 (2016).

**Publisher’s note:** Springer Nature remains neutral with regard to jurisdictional claims in published maps and institutional affiliations.

## Supplementary Material

Supplementary Information

## Figures and Tables

**Table 1:** General information of ribonucleotide reductase genes in “*Candidatus* Liberibacter asiaticus” A4 genome.

No.	Name	Location	Nucleotide (bp)	Amino acid	PI^a^
β-subunit					
1	*nrdB*^*S1*^	4924–5301	378	125	5.72
2	*nrdB*^*L1*^	67911–68970	1,059	352	5.56
3	*nrdB*^*L2*^	808799–809857	1,059	352	5.56
4	*nrdB*^*L3*^	955219–956277	1,059	352	5.56
5	*nrdB*^*S2*^	999494–999871	378	125	5.72
α-subunit					
1	*nrdA*	768834–771698	2,865	954	6.24

^a^PI is the protein isoelectric point.

**Table 2:** Selected bacterial whole genome sequences and their numbers of class Ia ribonucleotide reductase genes, α-subunit *nrdA* and β-subunit *nrdB*.

Bacterial name	Genome size (MB)	Accession no.	*nrdA*	*nrdB*	Representative *nrdB*^a^
“*Candidatus* Liberibacter asiaticus” str. A4	1.23	CP010804.1	1	5	WP_015452679.1/CD16_00035
“*Candidatus* Liberibacter asiaticus” str. Psy62	1.23	CP001677.5	1	5	WP_015452679.1/ACT57479.1
“*Candidatus* Liberibacter asiaticus” str. gxpsy	1.27	CP004005.1	1	5	WP_015452679.1/AGH17518.1
“*Candidatus* Liberibacter asiaticus” str. Ishi-1	1.19	AP014595.1	1	5	WP_015452679.1/BAP26775.1
“*Candidatus* Liberibacter asiaticus” str. HHCA	1.15	JMIL00000000.2	1	5	WP_031935117.1/ED07_RS0205390
“*Candidatus* Liberibacter asiaticus” str. FL17	1.23	JWHA00000000.1	1	5	KIH95982.1/RH08_RS00070
“*Candidatus* Liberibacter asiaticus” str. YCPsy	1.23	LIIM00000000.1	1	5	KPG63034.1/AL011_RS04410
“*Candidatus* Liberibacter asiaticus” str. SGCA5	1.20	LMTO00000000.1	1	3	NA
“*Candidatus* Liberibacter americanus” str. Sao Paulo	1.20	CP006604.1	1	1	AHA27773.1
“*Candidatus* Liberibacter americanus” str. PW_SP	1.18	AOFG00000000.1	1	1	WP_040055882.1
“*Candidatus* Liberibacter africanus” str. PTSAPSY	1.19	CP004021.1	1	1	WP_047264385.1
“*Candidatus* Liberibacter solanacearum” str. CLso-ZC1	1.26	CP002371.1	1	1	WP_044054292.1
“*Candidatus* Liberibacter solanacearum” str. R1	1.20	JNVH00000000.1	1	1	WP_034441434.1
“*Candidatus* Liberibacter solanacearum” str. HenneA	1.21	JQIG00000000.1	1	1	WP_034441434.1
“*Candidatus* Liberibacter solanacearum” str. LsoNZ1	1.31	JMTK00000000.1	1	1	WP_034441434.1
“*Candidatus* Liberibacter solanacearum” str. RSTM	1.29	LLVZ00000000.1	1	1	WP_034441434.1
*Liberibacter cresnt* BT-1	1.50	CP003789.1	1	1	WP_051012100.1
*Escherichia coli* O157:H7 str. Sakai	5.59	BA000007.2	2	1	NP_311145.1
*Agrobacterium tumefaciens* K84 chromosome 1	4.01	CP000628.1	1	0	NA
*Agrobacterium tumefaciens* K84 chromosome 2	2.65	CP000629.1	0	1	WP_012649863.1
*Pseudomonas stutzeri* A1501	6.59	CP000304.1	1	1	WP_011912798.1
*Xanthomonas axonopodis* pv. *citri* str. 306	5.27	AE008923.1	1	1	WP_005914812.1
*Mycoplasma hyopneumoniae* 232	0.90	AE017243.1	0	1	AAV27433.1
*Mycobacterium tuberculosis* H37Rv	4.41	AL123456.3	1	1	YP_177853.1

^a^When present, long form (left) and short form (right) are separated by “/”. The *nrdB* genes from different genomes are showed as the gene accession number or locus tag. NA = No annotation was found in NCBI GenBank nucleotide database (version 211.0). Partial sequences of *nrdB* genes from the genome of CLas strain SGCA5 were extracted for analyses.

**Table 3:** General information of PCR primers in this study.

Name	Type	Sequence (5′ → 3′)	Amplicon size (bp)	Gene	PCR format	Reference
RNRf	Forward	CATGCTCCATGAAGCTACCC	80 bp	*nrdB,* β-subunit of ribonucleotide reductase	Taqman, SYBR green	This study
RNRr	Reverse	GGAGCATTTAACCCCACGAA				
RNRp	Probe	FAM-CCTCGAAATCGCCTATGCAC-BHQ				
HLBas	Forward	TCGAGCGCGTATGCAATACG	76 bp	16S rRNA gene	Taqman, SYBR green	12
HLBr	Reverse	GCGTTATCCCGTAGAAAAAGGTAG				
HLB-P	Probe	FAM-AGACGGGTGAGTAACGCG-BHQ				
LJ900f	Forward	GCCGTTTTAACACAAAAGATGAATATC	99 bp	*hyvI/hyvII* of prophage	SYBR green	15
LJ900r	Reverse	ATAAATCAATTTGTTCTAGTTTACGAC				

**Table 4:** Evaluation of primer sets RNRf/RNRr (nrdB-based) and HLBas/HLBr (16S rRNA gene-based) on detection of “*Candidatus* Liberibacter asiaticus” using field samples collected from China and USA.

Geographical Origin	No. of strains	SYBR Green real-time PCR		P-value^a^ (RNRf/RNRf vs. HLBas/HLBr)	ΔCt (RNRf/RNRr-HLBas/HLBr)	Real-time PCR system
		RNRf/RNRr	HLBas/HLBr			
China						
Guangdong	88	20.75 ± 2.13	22.50 ± 2.44	<0.0001	−1.75 ± 0.43	MJ system
Lab in Guangdong	20	18.23 ± 2.00	19.68 ± 2.06	0.01708	−1.45 ± 0.33	MJ system
Yunnan	53	21.06 ± 1.95	22.55 ± 2.05	<0.0001	−1.49 ± 0.62	MJ system
Hainan	31	18.80 ± 1.17	20.24 ± 1.13	<0.0001	−1.44 ± 0.39	MJ system
Guangxi	7	20.95 ± 1.31	22.62 ± 1.25	0.03068	−1.67 ± 0.20	MJ system
Zhejiang	15	19.10 ± 1.77	20.68 ± 1.99	0.02954	−1.58 ± 0.60	MJ system
Jiangxi	13	20.67 ± 1.39	22.12 ± 1.58	0.01993	−1.46 ± 0.45	MJ system
Fujian	12	19.39 ± 1.57	20.96 ± 1.68	0.02733	−1.57 ± 0.17	MJ system
USA						
Florida	5	19.30 ± 1.01	20.90 ± 1.18	0.04989	−1.60 ± 0.18	ABI system
Texas	5	24.22 ± 0.99	26.40 ± 1.08	0.01047	−1.64 ± 0.05	ABI system
California	13	25.01 ± 1.94	26.38 ± 1.98	0.03090	−1.36 ± 0.44	MJ system
Total	262					
Mean Ct		20.27 ± 2.25	21.86 ± 2.32	<0.0001	−1.59 ± 0.46	

^a^Independent-sample T-test.
