# Supplementary material for: Unusual Five Copies and Dual Forms of nrdB in “Candidatus Liberibacter asiaticus”: Biological Implications and PCR Detection Application
Source: Sci Rep. 2016 Dec 13;6:39020. doi: 10.1038/srep39020 (PMC5154197; doi:10.1038/srep39020)
Supplement: Supplementary Information [file srep39020-s1.pdf]

Unusual Five Copies and Dual Forms of *nrdB* in “*Candidatus* Liberibacter asiaticus”: Biological Implications and PCR Detection Application

Zheng Zheng[1,2], Meirong Xu[1], Minli Bao[1], Fengnian Wu[1,2], Jianchi Chen[2]*, Xiaoling Deng[1]*

[1] Guangdong Province Key Laboratory of Microbial Signals and Disease Control, Citrus Huanglongbing Research Laboratory, South China Agricultural University, Guangzhou, Peoples' Republic of China

[2] San Joaquin Valley Agricultural Sciences Center, United States Department of Agriculture – Agricultural Research Service, Parlier, California, United States of America

*Correspondent authors: E-mail: xldeng@scau.edu.cn (X. D.) / jianchi.chen@ars.usda.gov (J. C.)

Supplementary Table S1. Annotated information of repeat DNAs in "*Candidatus* Liberibacter asiaticus" strain A4 corresponding to Fig. 1.

Region	Sequence position	Size (bp)	Open Reading Frames (ORFs)/Genes									
			nrdB^{L}		Hypothetical protein	16S rRNA	tRNA-Ile	tRNA-Ala	23S rRNA	5S rRNA	tRNA-Met	Hypothetical protein
			-	*nrdB*^{S}								
1	3422-5299	1881	-	CD16_00035	CD16_00030	-	-	-	-	-	-	-
2	67911-68970	1059	CD16_00300		-	-	-	-	-	-	-	-
3	406600-412372	5769	-	-	-	CD16_01840	CD16_01835	CD16_01830	CD16_01825	CD16_01820	CD16_01815	CD16_01810
4	779815-785583	5769	-	-	-	CD16_03520	CD16_03525	CD16_03530	CD16_03535	CD16_03540	CD16_03545	CD16_03550
5	808799-809857	1059	CD16_03625		-	-	-	-	-	-	-	-
6	847796-853564	5769	-	-	-	CD16_03795	CD16_03800	CD16_03805	CD16_03810	CD16_03815	CD16_03820	CD16_03825
7	885003-886491	1491	-	-	CD16_03980	-	-	-	-	-	-	-
8	909207-910695	1491	-	-	CD16_04055	-	-	-	-	-	-	-
9	955219-956277	1059	CD16_04230		-	-	-	-	-	-	-	-
10	999496-1001374	1881	-	CD16_04445	CD16_04450	-	-	-	-	-	-	-

Supplementary Table S2. Evaluation of primer sets RNRf/RNRr (nrdB-based) and HLBas/HLBr (16S rRNA gene-based) on detection of "*Candidatus* Liberibacter asiaticus" by using different Real-time PCR system. *Single factor ANOVA (Duncan's multiple range test) at 95% (P = 0.05) confidence interval was used to determine statistical significance of three data sets. The same column with different letters represent significant difference.

Real-time PCR System	Total No. of isolates	SYBR Green real-time PCR		ΔCt* (RNRf/RNRr-HLBas/HLBr)	P value
		RNRf/RNRr	HLBas/HLBr		
ABI system	57	20.05 ± 2.22	21.73 ± 2.24	-1.68 ± 0.18a	<0.0001
MJ system	239	20.38 ± 2.14	21.98 ± 2.20	-1.60 ± 0.49ab	<0.0001
CFX system	20	18.23 ± 2.00	19.68 ± 2.06	-1.45 ± 0.33b	0.0296
Average (St. dev.)		20.24 ± 2.34	21.79 ± 2.41	-1.55 ± 0.65	<0.0001

Supplementary Table S3. Two genes up- and down-stream of nrdB in the genome of "*Candidatus* Liberibacter asiaticus" strain A4.

No.	- 2 ORF	- 1 ORF	Intergenic (bp)	RNR gene	Intergenic (bp)	+ 1 ORF	+ 2 ORF
β-subunit							
1	Integrase	Hypothetical protein	160	*nrdB*^{S1}	1268	Malate dehydrogenase	ABC transporter
2	Glutaminase	30S ribosomal protein S4	839	*nrdB*^{L1}	941	Fumarate hydratase	Alanyl-tRNA synthetase
3	Glucose transporter	Hypothetical protein	172	*nrdB*^{L2}	119	Riboflavin kinase	HAD family hydrolase
4	Hypothetical protein	Hypothetical protein	145	*nrdB*^{L3}	129	Hypothetical protein	Facilitator transporter
5	Threonine-tRNA ligase	Hypothetical protein	185	*nrdB*^{S2}	160	Hypothetical protein	Hypothetical protein
α-subunit							
1	Glucose-6-phosphate dehydrogenase	Glutamine synthetase	1056	*nrdA*	82	Hypothetical protein	Hypothetical protein
